# Enhanced Integrated Behavioral Health Model Improves Depressive Symptoms in a Low-Income, Uninsured, Primarily Hispanic Population Served by a Free and Charitable Clinic

**DOI:** 10.5334/ijic.5421

**Published:** 2020-11-24

**Authors:** Amy Flynn, Erika Gaitan, Rebecca Stocker, Elizabeth Showalter, Karen Sautter Errichetti

**Affiliations:** 1 Health Resources in Action, Boston, MA, US; 2 Hope Family Health Center, McAllen, TX, US

**Keywords:** integrated behavioral health, depressive symptoms, RCT, Hispanic, uninsured, border region, free clinic

## Abstract

**Background:**

Residents living in US-Mexico border communities have some of the worst health outcomes nationally. This randomized trial evaluated whether patients receiving enhanced integrated behavioral health (IBH) care at a southern Texas free and charitable were more likely to improve health outcomes after 12 months compared to patients receiving standard care.

**Theory & Methods:**

The IBH intervention featured brief intervention by a behavioral health specialist and enhanced coordinated care. The primary outcome was systolic blood pressure. Secondary outcomes were diastolic blood pressure, HbA1c, BMI, and depressive symptoms. Linear regression models were utilized to assess the impact of IBH on participants; secondary analyses examined possible effect modification.

**Results:**

After 12 months, intervention participants (n = 172) were more likely to have a lower PHQ-9 score than control participants (n = 198) (β = –1.67, p = 0.01). There was significant modification of the intervention effect by age; there was a different effect on older participants (β = –2.08, p = 0.01). There were no statistically significant findings for other outcomes.

**Conclusions:**

Collaborative, integrated behavioral health and primary care can improve depressive symptoms for low-income or uninsured individuals living in southern Texas border communities. These findings provide evidence that may help develop IBH programs to improve health of vulnerable populations experiencing health inequities.

## Introduction

Residents of the Rio Grande Valley (RGV), Texas have among the worst health outcomes nationally. Rates of chronic disease such as obesity, diabetes, and related mortality in the RGV exceed those in most other regions of Texas and the nation [1,2]. Poverty is pervasive, placing residents at high risk for poor health; they are more likely to be exposed to environmental hazards and have higher rates of chronic physical and mental health concerns [3,4,5].

At its foundation, integrated behavioral health care (IBH) aims to address multiple health concerns, and related social determinants, by bringing behavioral health and primary care services together. No single model of IBH can be applied universally to health or social service settings; however, each approach is team-based and involves collaboration between multidisciplinary health and social service providers to achieve shared patient and community outcomes [6]. The SAMSHA-HRSA Center for Integrated Health Solutions categorizes these approaches into three levels: coordinated, focused on communication between providers; co-located, models bringing different services into the same physical space; and integrated, which involves practice and workflow changes [7]. A 2016 update on research on integration of behavioral health and primary care emphasized the increasing strength of evidence highlighting the success of these approaches [8]. IBH has been effective in various populations [9,10,11,12] with some studies focusing on specialty populations such as adolescents and those with substance use disorders or severe mental illness, which may need tailored and focused integrated care [13,14,15].

A growing body of evidence demonstrates IBH care approaches improve population health and access to health care in areas similar to South Texas [16,17,18]. In Austin, Texas for example, People’s Community Clinic used an IBH model to enable adult primary care patients diagnosed with depression and anxiety to receive psychiatric medication, counseling, and education. The program achieved results typically seen only in controlled clinical trials. The IBH model improved patients’ mental health outcomes with a minimal investment of resources [19]. Similarly, Bridges et al. revealed that Latinos participating in IBH care had significant improvements in symptoms and expressed high satisfaction with IBH treatment [20].

Despite the promise of integrated care strategies in improving physical and mental health, key gaps exist in evidence of the generalizability of these approaches in diverse populations. To the authors’ knowledge, no randomized control trials (RCT) of integrated care approaches have been conducted in free and charitable clinic settings or in clinics exclusively engaging volunteer primary care providers. Moreover, literature describing the impact of integrated care strategies on primarily Hispanic populations living in poverty at the US-Mexico border is sparse.

Hope Family Health Center (HOPE), a nonprofit free and charitable clinic in McAllen, Texas, provides medical and behavioral health services to uninsured patients living at or below the 200% federal poverty level (FPL). HOPE’s primary care providers are exclusively volunteers. In order to improve patient physical and mental health outcomes, HOPE adapted the collaborative care model to its clinic operations [19,21]. HOPE aimed to remove barriers between behavioral and primary care through service co-location and integration supported by care management. Without effective intervention, it is likely individuals living in HOPE’s service area would not receive timely integrated care due to regional healthcare disparities, poverty, and lack of insurance.

In this RCT, we evaluated the effectiveness of HOPE’s integrated care model in a predominantly Hispanic, uninsured population receiving services from a free and charitable clinic located near the US-Mexico border. We hypothesized that integrated care would reduce blood pressure, HbA1c, body mass index, and depressive symptoms among adult patients with chronic illness living at or below the 200% FPL.

## Theory & Methods

### Study Setting & Design

HOPE provides free medical, counseling, and case management services to over 1,800 uninsured residents in the RGV annually, an area where almost one-third of the population are living in poverty [22]. The clinic is staffed with volunteer primary care providers and employed behavioral health counselors. Prior to the study, HOPE had co-located primary care and behavioral health services in the same physical space but had not fully integrated services through care coordination within that space.

We conducted an RCT to compare outcomes of intervention participants receiving HOPE’s IBH model with control participants receiving usual care. Participants enrolled in the study were followed for approximately 12 months and assessed for physical and behavioral health outcomes at baseline, 6- and 12-months. HOPE concurrently studied the implementation of their model as part of a separate research study [23].

### Study Participants

All HOPE patients are uninsured and living at or below 200% of the FPL as eligibility criteria for either primary care or behavioral health services at the clinic. HOPE patients were eligible to participate in the study if they were receiving behavioral health services from HOPE at the time of enrollment; resided in Cameron, Hidalgo, Willacy, or Starr County; and had a diagnosis of one or more chronic conditions: hypertension (blood pressure ≥140/90 mmHg), obesity (body mass index ≥30.0), poorly controlled diabetes (HbA1c ≥ 6.8%), or moderate depression (Patient Health Questionnaire (PHQ-9) score ≥10).

HOPE identified potential participants by pre-screening their data system for eligibility criteria. Patients flagged as potentially eligible were then assessed for eligibility in person at their next visit. Patients meeting the eligibility criteria were invited to participate in the study. Those who agreed to participate underwent an informed consent process including the study overview, information about the randomization process, and an understanding that voluntary participation included taking a baseline and all follow-up assessments. The study research protocol was approved by New England Independent Review Board (Reference #120160447).

**Figure 1:**
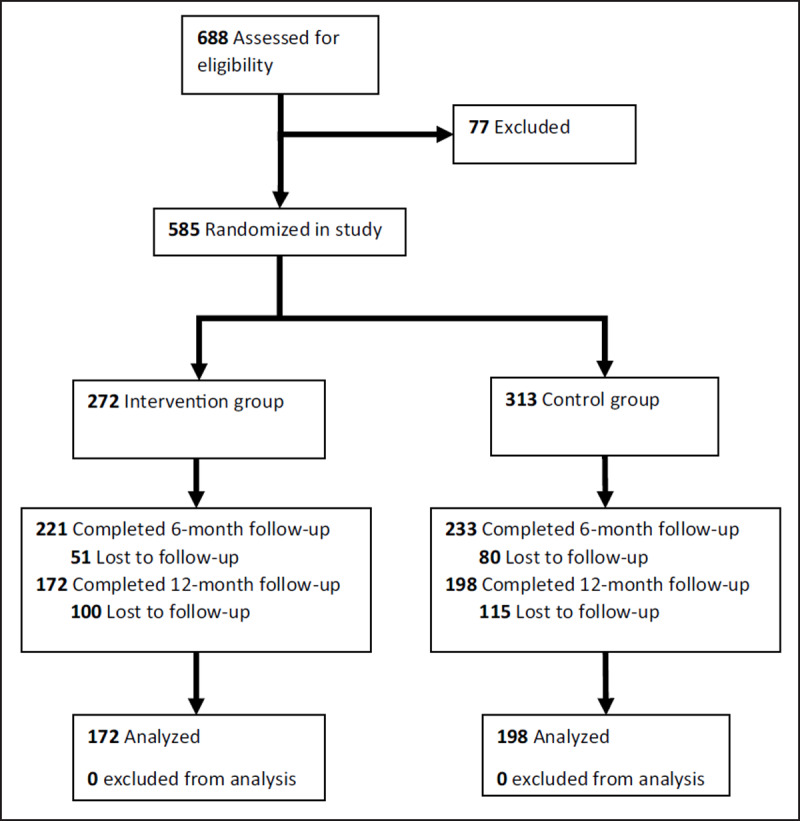
Study Flow Diagram.

688 patients were identified as eligible to participate via pre-screening of records; 585 agreed to participate when approached by clinic staff and were allocated 1:1 to the intervention and control groups. Patients were excluded if they were pregnant or did not meet the eligibility criteria (Figure 1).

### Randomization and Baseline Assessment

After the informed consent process, participants were allocated to the intervention or control group through simple random assignment using a computer-generated random number calculator and participant selection of number from individual, unmarked envelopes. After assignment, participants were seen by a nurse manager to complete baseline assessment, including the PHQ-9. A medical assistant assessed vitals including height, weight, and blood pressure. Baseline sociodemographic data were collected from HOPE’s data system. Two follow-up assessments were conducted in person when participants attended medical appointments to collect subsequent health data, at 6 months (midpoint) and 12 months (endpoint) after enrollment.

HOPE intended for intervention participants to receive care from a group of volunteer primary care providers separate from those treating the usual care participants. These individuals were to participate in the integrated program serving as “intervention only” providers. In practice, providers saw both intervention and control participants. This shift in design was primarily due to capacity of providers and HOPE’s priority to ensure patient care continuity. Additionally, volunteer providers’ variable schedules posed a challenge to implementing this aspect of the study design.

### Integrated Behavioral Health Intervention

**Figure 2:**
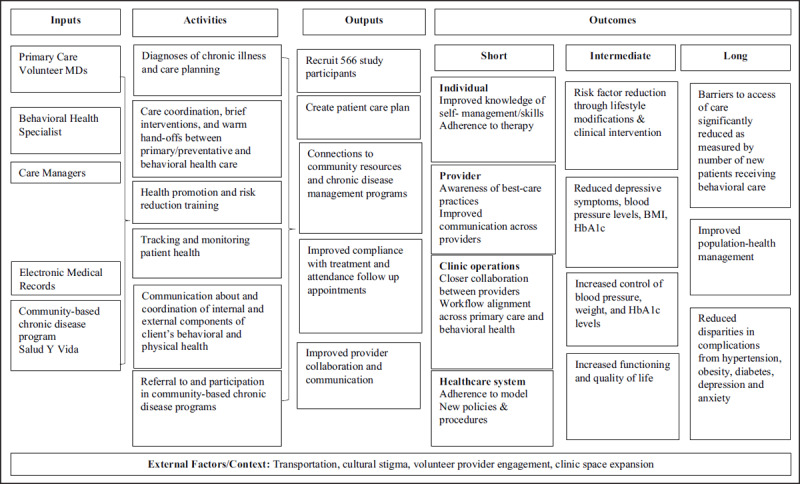
HOPE IBH Program Logic Model.

HOPE adapted the collaborative care model in December 2015 (Figure 2) [19,21,24,25]. HOPE’s approach included activities from the Sanchez and Watt model that have been linked to improved health outcomes, including care management and access to behavioral health specialists [19]. In addition, HOPE’s program activities were adapted for their unique border community, including bilingual programming and psychoeducation.

Participants randomized to the intervention group received enhanced integrated primary care and behavioral health services (Table 1). As part of the intervention, participants received a care plan developed collaboratively by providers of different services. These plans included at least one visit with a behavioral health specialist (BHS) and chronic care management visits with a transitional nurse. The addition of the BHS and transitional nurse enhanced the behavioral health services previously available at HOPE by integrating these services into the primary care workflow. The BHS conducted assessments, provided initial counseling, and coordinated referrals to care management and/or community-based health services. The BHS also made warm-handoffs as appropriate (e.g. to primary care or additional behavioral health counseling). The chronic care management visits focused on enabling patients to understand their chronic conditions (e.g. diabetes, obesity) and strategies to managing these conditions. The transitional nurse served as an additional resource to services addressing nutrition, exercise, smoking cessation, and support groups. Intervention participants also received services from a care coordinator who made warm-handoffs to either the BHS, transitional nurse, internal pharmacy services, or coordinated referrals to other support services available in the community.

**Table 1:** Availability of Services for Usual and Integrated Care Participants.

	HOPE Usual Care	HOPE Integrated Care

Coordinated information sharing (e.g. development of care plans, record keeping)		✓
Primary care services	✓	✓
Individual and group behavioral health counseling services	✓	✓
Behavioral Health Specialist (BHS) assessments		✓
BHS initial individual counseling and follow-up sessions		✓
Chronic care management visits (with transitional nurse)		✓
Referrals to social and health education services	✓	✓
Coordination of referrals to external social and health education services		✓
Warm hand-offs between services within HOPE		✓

### Usual Care

Control group participants received HOPE’s usual care including primary care services and behavioral health counseling co-located within the clinic, but unlike the intervention participants, they were not offered the enhanced services from the transitional nurse or BHS. Control participants could be referred to additional specialty and community-based services. However, external referrals were not coordinated by the care coordinator and these participants did not receive warm-handoffs to other HOPE providers from the care coordinator.

### Outcomes, Covariates, and Modifiers

The primary outcome for this study was blood pressure as measured by a medical assistant using a manometer [26]. Secondary outcomes were HbA1c (a measure of glucose control), body mass index (BMI), and depressive symptoms (assessed through PHQ-9 score) [27]. The PHQ-9 assessment was administered through provider interview. HbA1c was measured by blood test [28]. BMI was measured using a clinical scale and height tool [29].

We collected participant sociodemographic measures at baseline including age, sex (male; female), ethnicity (Hispanic; non-Hispanic), employment (employed; unemployed), marital status (married; not married, which included those who were divorced, partnered, separated, single, or widowed), and primary language spoken (English or Spanish). Clinic administrative data regarding the number and type of visits received by each participant were collected.

### Statistical Analysis

Our primary analyses used an intent-to-treat approach to assess the difference in health outcomes between individual participants in the intervention and control groups at 12 months. T-tests were conducted to test within-group differences across time (baseline to endpoint) and between groups at study baseline and endpoint for each of the continuous outcomes. Chi square tests were used to assess equivalence at baseline. We then estimated multivariate linear regression models adjusting for important covariates and potential confounders identified in the assessment of baseline equivalence and bivariate analyses using backward elimination model selection for parsimony [30]; covariates were removed from the model where p > 0.15. Pearson correlation coefficients were calculated to identify collinearity among variables in each model. Effect sizes for statistically significant results were calculated using the Cohen’s d statistic. We subsequently explored the potential for effect modification by baseline health condition (e.g., baseline depression as an effect modifier for impact on PHQ-9 score at 12 months), age (below versus equal to or above the mean study age of 51 years), and sex. Stratified analyses were performed if significant interaction terms were detected. All analyses were conducted using SAS version 9.4 (Cary, NC), and differences where p < 0.05 were considered statistically significant.

All participants enrolled had complete baseline sociodemographic data collected. While efforts were made to collect full data for outcome measures at baseline, there were some challenges. Missing data for outcome measures blood pressure, BMI, and depressive symptoms ranged 1–21% at baseline and 11–19% at 12 months. Prior to analysis, we multiply imputed the missing outcome data with the mi procedure in SAS using all available baseline sociodemographic and health data, generating 10 datasets. Analyses of these four outcomes employed the MIANALYZE procedure in SAS. Because HbA1c level was not universally collected from participants, multiple imputation was not applied to the primary model of this outcome.

We conducted separate sensitivity analyses to assess for differential attrition by study group and potential contamination of intervention effect. We compared the proportion of participants who were lost to follow-up in the intervention versus control groups using a Chi-square test. Due to clinical staff and volunteer providers treating both intervention and control participants, there was concern about contamination of the control group. Thus, sensitivity analyses using a per protocol approach were conducted to verify the validity of results found in the intent-to-treat analyses following the approach described above. Clinic administrative data regarding visits with various providers were used to understand the extent participants received the level of care to which they were randomly assigned. For these analyses, intervention receipt was operationally defined as any evidence of care coordination in the medical record. Control participants with evidence of having received care coordination (n = 38) and intervention participants with no evidence of care coordination (n = 45) were removed from the sample for these sensitivity analyses.

## Results

### Baseline Participant Characteristics

Most participants were female (73.5%) and Hispanic (83.2%) with a mean age of 51 years. No statistically significant differences were identified between the intervention and control groups in terms of observed sociodemographic measures or health outcomes at baseline, except for PHQ-9 score (Table 2). The intervention group had a significantly higher mean PHQ-9 score (7.1) than the control group (5.0) at baseline (p = 0.001).

**Table 2:** Study Participant Characteristics at Baseline by Intervention Group.

	Intervention (n = 270)		Control (n = 312)		p-value

	n	%	n	%	

*Demographics*					
Sex					
Female	199	73.7	229	73.4	0.93
Male	71	26.3	83	26.6	
Ethnicity					
Hispanic	217	80.4	267	85.6	0.09
Non-Hispanic	53	19.6	45	14.4	
Age, mean (SD)	51.2 (10.3)	—	50.6 (10.7)	—	0.51
Employment Status					
Employed	2	0.7	5	1.6	0.34
Unemployed	268	99.3	307	98.4	
Marital Status					
Married	135	50.0	161	51.6	0.70
Not Married	135	50.0	151	48.4	
Primary Language Spoken					
English	28	10.4	40	12.8	0.36
Spanish	242	89.6	272	87.2	
*Health, mean (SD)*					
Systolic Blood Pressure	133.4 (21.3)		134.5 (19.4)		0.48
Diastolic Blood Pressure	81.0 (9.6)		82.0 (9.2)		0.17
BMI	33.9 (7.5)		33.6 (6.6)		0.85
PHQ-9 score	7.1 (7.1)		5.0 (6.3)		0.001
HbA1c	8.0 (2.5)		7.6 (2.3)		0.12

370 participants completed a 12-month follow-up assessment; 172 intervention and 198 control. The intervention and control groups had the same rate of retention (63%; p = 0.95), indicating no differential attrition.

### Intent-to-Treat Analysis

In adjusted analyses, the intervention and control groups differed significantly on depressive symptoms at 12-months; the groups were similar on HbA1c, blood pressure, and BMI (Table 3). On average, those in the intervention group had a PHQ-9 score that was 1.67 points lower than those in the control group (*p* = 0.01, *d* = 0.29).

**Table 3:** Effect of IBH Intervention on Health Outcomes at 12 Months, Intent-to-Treat Analyses.

	n	Intervention *Mean (SD)*	Control *Mean (SD)*	Intervention – Control *Adjusted Mean Difference (SE)*	p-value

Systolic Blood Pressure^a^	370	128.4 (18.3)	130.0 (20.3)	–2.47 (1.70)	0.15
Diastolic Blood Pressure^b^	370	78.1 (7.1)	79.0 (8.5)	–0.93 (0.75)	0.22
HbA1c^c^	146	8.0 (1.9)	7.7 (1.9)	–0.11 (0.24)	0.67
BMI^d^	370	34.3 (7.7)	33.1 (6.9)	0.14 (0.22)	0.52
PHQ-9 score^e^	370	4.6 (5.4)	5.3 (6.2)	–1.67 (0.66)	0.01

^a^ adjusted for age, sex, marital status, baseline systolic blood pressure, and baseline PHQ-9 score ^b^ adjusted for marital status, baseline diastolic blood pressure, and number of comorbidities at baseline ^c^ adjusted for baseline HbA1c level ^d^ adjusted for baseline BMI and baseline PHQ-9 score ^e^ adjusted for age, primary language, employment status, and baseline PHQ-9 score.

### Per-Protocol Analysis

The results of sensitivity analyses were like the intent-to-treat analyses (Table 4). No statistically significant effects were detected for physical health measures; additionally, intervention participation was associated with a mean PHQ-9 score 1.64 points lower (p = 0.04) in the per protocol subsample.

**Table 4:** Effect of IBH Intervention on Health Outcomes at 12 Months, Per Protocol Analysis.

	n	Intervention – Control *Adjusted Mean Difference (SE)*	p-value

Systolic Blood Pressure^a^	285	–1.11 (1.87)	0.55
Diastolic Blood Pressure^b^	285	–0.77 (0.76)	0.33
HbA1c^c^	102	–0.23 (0.28)	0.42
BMI^d^	281	–0.01 (0.23)	0.97
PHQ-9 score^e^	188	–1.64 (0.74)	0.04

^a^ adjusted for age, marital status, baseline systolic blood pressure, and number of comorbidities at baseline ^b^ adjusted for age, baseline diastolic blood pressure, and number of comorbidities at baseline ^c^ adjusted for baseline HbA1c level ^d^ adjusted for baseline BMI ^e^ adjusted for baseline PHQ-9 score.

### Effect Modification

Because primary and sensitivity analyses produced similar results, effect modification analyses were conducted using the intent-to-treat sample. Significant interactions were detected between sex and study group in analyses of 12-month blood pressure measures and HbA1c level. In sex-stratified models, the intervention was not statistically associated with blood pressure or HbA1c level for either sex (not shown). A significant interaction between study group and age was detected for 12-month PHQ-9 scores. In age-stratified models, intervention participants aged 51 years or older had a PHQ-9 score 2.08 points lower than those in the control group (*p* = 0.01, *d* = 0.34, Table 5) on average; however, the intervention effect was not significant among participants under 51 years of age. There were no significant interactions of study group and potential effect modifiers for 12-month BMI.

**Table 5:** Effect of IBH on PHQ-9 Score at 12 Months, Stratified by Mean Age Group, Intent-to-Treat Analysis.

	n	Intervention – Control *Adjusted Mean Difference (SE)*	p-value

Participants 51+ Years^a^	206	–2.08 (0.81)	0.01
Participants Under 51 Years^a^	164	–1.34 (0.88)	0.13

^a^ adjusted for baseline PHQ-9 score.

## Discussion

This trial demonstrated that a behavioral health and care coordination intervention implemented in a free and charitable clinic with voluntary care providers can reduce depressive symptoms in uninsured patients living at or below the 200% FPL compared to standard care. When controlling for baseline measures and other covariates, intervention participants had significantly reduced PHQ-9 score at 12-months compared to control participants. These results align with findings from previous studies of IBH and its effect on mental health [31,32]. The study also found evidence of effect modification by participation age, with greater impact identified among older intervention participants. This result has the potential to respond to the literature that has identified difficulties in treating depression in older adults and the need for approaches targeting symptoms in an older population [33,34].

The finding that depressive symptoms changed but physical outcomes such as blood pressure did not is interesting and notable. HOPE’s integrated care approach emphasized brief intervention by the BHS and referral to counseling, which may be one mechanism by which depressive symptoms were mitigated. However, it is not surprising that physical outcomes did not change. First, there may have been insufficient time to observe a difference given the single year of follow up, and it is possible that physical changes may have occurred over a longer intervention period. Second, literature suggests that patients on anti-depressant drugs have increased risk of cardiovascular disease particularly if there is pre-existing cardiovascular disease [35]. However, we were unable to assess the impact of pharmacological intervention due to challenges in obtaining information about medications.

These findings fill an important gap in the integrated care literature by demonstrating the impact of behavioral health service integration with primary care in a previously understudied population. To our knowledge, this is the first RCT to examine an adapted integrated behavioral health model within the unique service and population characteristics of our context: a free and charitable clinic setting reliant on volunteer clinical providers serving uninsured predominately Hispanics living in poverty at the US-Mexican border. Our results demonstrate the positive impact integrated care can have on health outcomes.

The effects demonstrated in the trial occurred despite a lack of feasibility to implement two planned program components: psychiatry consultation and volunteer primary care provider engagement. Results suggest that psychiatric consultation was not necessary to improve depressive symptoms in a charitable clinic setting. Professional shortages are common in areas served by free and charitable clinics, and engagement of mental health professionals in an IBH intervention may improve feasibility and sustainability of the approach; however, additional research is needed to validate this finding and guide other clinics working to provide similar programming. Similarly, HOPE planned to engage volunteer primary care providers more directly in the intervention, but the episodic character of provider work at the clinic made this impossible. While the trial significantly improved mental health despite this, it is possible that physical health outcomes may have improved with more direct engagement of these providers.

The study has several limitations. Contamination occurred within the trial due to implementation challenges and the single clinic setting. However, sensitivity analyses suggested the impact of contamination was minimal and did not bias study results. Additionally, receipt of services outside of HOPE was not tracked and therefore not considered in these analyses. A limited number of patient characteristics were available for equivalence testing, and unmeasured differences between groups is possible. However, covariates included in our final models encompass biological and social factors known to be influential for health outcomes; additionally, randomization was successful based on the measured covariates, supporting the credibility of our results. Retention rates for both groups were lower than anticipated which may have limited our ability to see effects in some outcomes; however, no differential attrition was detected. The unique characteristics of our population and service setting may limit the generalizability of these results.

In conclusion, implementing an integrated care model with a focus on collaboration and communication between primary care and behavioral health providers in a free and charitable clinic setting can improve depressive symptoms after 12 months for individuals who are low-income or uninsured living in the border region of southern Texas. The results from this study can have funding implications when decisions on settings for IBH, or other innovative care models, are being made. Cost-effectiveness studies of HOPE’s approach would be very useful in understanding the feasibility of such models in similar clinics.
